# Exercise for Stroke Rehabilitation: A Bibliometric Analysis of Global Research From 2001 to 2021

**DOI:** 10.3389/fnagi.2022.876954

**Published:** 2022-06-17

**Authors:** Yulin Dong, Linman Weng, Yinhu Hu, Yuxing Mao, Yajuan Zhang, Zefeng Lu, Tingting Shi, Renren Du, Wu Wang, Jinyan Wang, Xueqiang Wang

**Affiliations:** ^1^Department of Treatment, The Second Rehabilitation Hospital of Shanghai, Shanghai, China; ^2^Department of Sport Rehabilitation, Shanghai University of Sport, Shanghai, China; ^3^Department of Rehabilitation Medicine, Shanghai Shangti Orthopaedic Hospital, Shanghai, China

**Keywords:** stroke, rehabilitation, bibliometric analysis, exercise, global trend

## Abstract

**Objective:**

To make a bibliometric analysis of global trends in research into exercise interventions for stroke between 2001 and 2021.

**Method:**

This study did the systematic literature from 2001 to 2021 in Web of Science Core Collection. CiteSpace software was used to analyze the relationship of publications with countries, journals, authors, references, and keywords.

**Results:**

A total of 3,484 publications were obtained in the bibliometric analysis. The number of publications increased gradually over the period. The United States have the most number of publications. The journal *stroke* had the most citations per paper (106.95) and the highest impact factor (IF 2020, 7.194). The most high frequency keywords are “stroke,” “rehabilitation,” and “recovery,” the top of burst key words are “health,” “speed,” and “aerobic exercise”.

**Conclusion:**

These findings provide the trends of exercise for stroke s and provided the potential research frontiers in the past 20 years. It will be a useful basis for further research into focus issues, cooperators, development trends.

## Introduction

Stroke is a kind of cerebrovascular diseases, and its main clinical manifestations are cerebral ischemia and hemorrhagic injury. In 2017, there were about 104.2 million stroke survivors and 11.9 million new cases of stroke worldwide (Gbd 2017 Disease and Injury Incidence and Prevalence Collaborators, 2018). On a global scale, stroke is the second leading cause of death and the third leading cause of disability (Johnson et al., 2016). The prevalence of stroke and mental illnesses is estimated to result in continued economic cost worth $240.67 billion by 2030 (Ovbiagele et al., 2013). Stroke not only affects the long-term functional disability but also increases the economic burden on families and the healthcare system (Prince et al., 2015). Therefore, low-cost and easily accessible treatments with minimal side effects must be developed to address stroke.

According to the American Heart Association/American Stroke Association, exercise therapy is beneficial to stroke survivors (Ovbiagele et al., 2013). Many studies have reported that gait-oriented training, aerobic treadmill training, intensive mobility training, and physiotherapeutic interventions, can improve disability for stroke survivors (Hesse et al., 2003; Eng and Tang, 2007; van de Port et al., 2007; Hesse, 2008; De Rooij et al., 2016). Several meta-analyses focus on specific exercise therapy for stroke, such as Tai Chi exercise (Wu et al., 2018), aerobic exercise (Wang et al., 2019), sling exercise (Chen et al., 2016), and traditional Chinese exercise (Chen et al., 2015). Various exercise therapies are used to treat stroke, but there is no clear evidence to support the superiority of any particular exercise intervention. Also, there are lack of systematic analysis of global research into exercise for stroke. Therefore, there is a need to summarize the progress of research into exercise therapy for stroke over recent decades, and to provide objective data on which to base future research.

Bibliometric analysis has been considered as a quantitative statistical tool to describe the knowledge structure and keyword trends in specific research areas (Hicks et al., 2015). This type of study could offer readers the general aspects of research topic on distribution by country, institution, author, and journal. Bibliometric methods have been used in many research fields, including stroke (Feng et al., 2013) and exercise (Fares et al., 2017; Khatra et al., 2021). From the perspective of research progress, the number of clinical trials and papers on stroke rehabilitation has increased in the past years (Feng et al., 2013). Exercise as a therapeutic intervention in the treatment, rehabilitation, and prevention of illness (McCrory, 2006). However, no detailed bibliometric analysis has been conducted on the topic of exercise for stroke.

To address the shortage of quantitative analysis of exercise for stroke research, the present study aims to obtain the global scientific outputs of the research using exercise from 2001 to 2021 and quantitative information about the distribution of countries, institutions, journals, authors and visual information. This study will allow researchers to understand the hotspots and emerging trends of research on exercise and stroke.

## Methods

### Search Strategy and Data Acquisition

Web of Science (WoS) contains 20,000 high-quality and influential academic journals related to 250 disciplines around the world. The database with comprehensive citation index records is conductive to data mining and co-citation analysis (Waqas et al., 2020). It is the authoritative data source for data extraction in bibliometric analysis, and has been used in many previous studies (Chen et al., 2012; Ellegaard and Wallin, 2015; Yan et al., 2020). It also had more accurate document type labeling than PubMed, Scopus (Yeung, 2019).

Publications on stroke and exercise research from 2001 to 2021 were retrieved and downloaded from the Science Citation Index Expanded (SCI-E) of WoS Core Collection (WoSCC). The search strategy was as follows: TI = (stroke OR apoplexy OR cerebrovascular accident OR cerebral hemorrhage OR hematencephalon OR encephalorrhagia OR cerebral ischemia) AND TI = (exercise OR train OR physical activity OR sport OR movement OR motion), language = English, and document type = article or review. A total of 3,484 publications were retrieved on 1 January 2022.

### Knowledge Visualization Analysis

Publication records, including countries or regions, journals, authors, keywords, references, citations, citations per year, H-index, and impact factor (IF), were extracted from WoSCC. The H-index is a numerical indicator that evaluates the impact of scholars’ or journals’ publications and performance (Hirsch, 2005). IF reflects the average number of citations of a paper published in a journal and assesses the relative importance of the journal (Sharma et al., 2014). CiteSpace V was used in building knowledge network and citation paths (Chen et al., 2019). The publication records from WoSCC were input into CiteSpace, which generated collaborative networks (countries and authors), co-occurrence networks (keywords), and co-citation networks (references). The node represents the subject analyzed. Larger nodes denote greater number of occurrences or citations. A centrality index greater than 0 will be marked with a purple ring around the node, representing landmark works or theories (Waqas et al., 2020). Burst keywords or references indicates that the subjects frequently appear or are cited over a period, and represents those relevant topics have attracted considerable attention from researchers. Thus, they can be regarded as a research hotspot or research frontiers (Chen, 2006). Important themes, recent developments, and emerging trends in the field can be detected effectively based on these cues. The relationship between the publication outputs and IF was analyzed by Pearson correlation analysis in IBM SPSS Statistics 26. Statistical significance was considered at *P* < 0.05.

## Results

### Publication Activity

**FIGURE 1:**
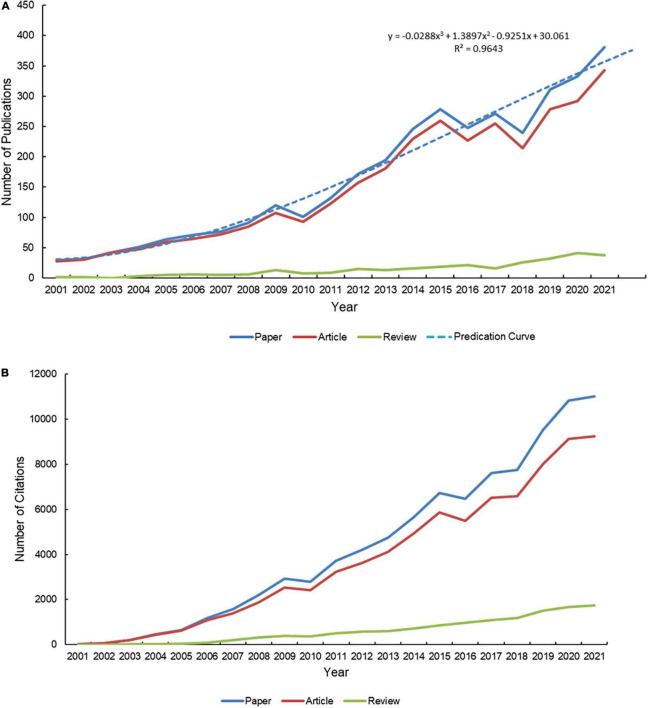
Trends of publications and citations. **(A)** Trends of publication outputs and growth prediction from 2001 to 2021. **(B)** Trends of citations received by publications from 2001 to 2021.

Web of Science Core Collection yielded 3484 publications during the period of 2001–2021, with a total H-index of 125 and citations of 90,348 by citing 39,995 papers. Each paper was cited 25.93 times on average. In general, annual papers and citations presented a growing trend. The average number of citations per paper in WoSCC was 0.34 in 2001 and 28.88 in 2021. The vast majority of these papers were articles. In 2021, the number of articles was 343 and received 9,248 cumulative citations, about 9.03 and 5.27 times more than reviews, respectively (Figure 1).

### Regional Trends

**FIGURE 2:**
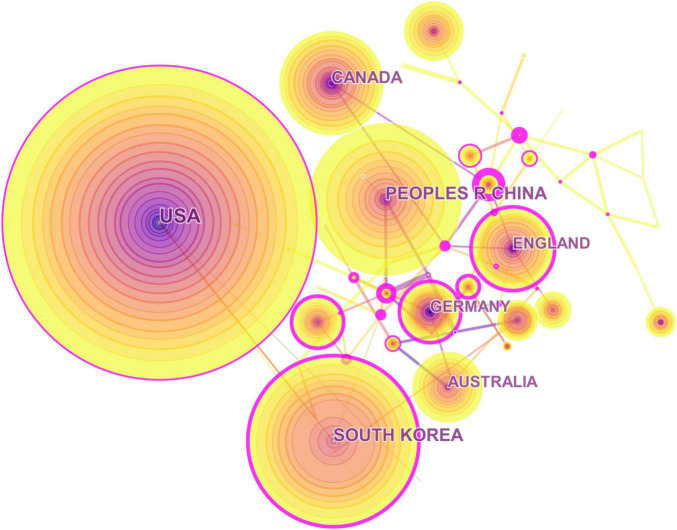
Collaboration network of countries or regions.

**FIGURE 3:**
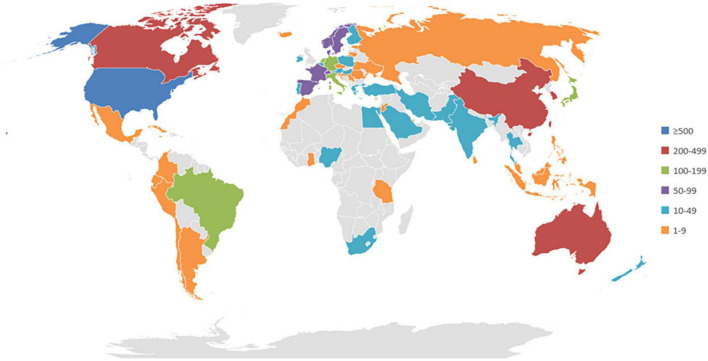
World map of the total output of countries or regions.

In accordance with the address of authors, 81 countries or regions contributed to these publications. The regions with the highest output of publications were in the United States, South Korea, China, Canada, and United Kingdom. Amongst the top 10 publication outputs, only China was a developing country, and others were developed countries. Switzerland, Austria, Singapore, Denmark, and Saudi Arabia were the top five countries and had a significant influence on global cooperation. These central nations are mainly developed countries, and they have also formed cooperative relations with several developing countries. In terms of publication output, none of the top five countries were considered important in the collaboration network (Figures 2, 3).

### Journal Characteristics

The papers were published in 584 academic journals. In the top 10 journals for publication output, *Journal of Physical Therapy Science* had the highest number of papers (175 publications), whereas *Disability and Rehabilitation* had the lowest number of papers (77 publications). Half of the journals were established in the United States, and all journals were based in developed countries. *Stroke* had the highest number of citations, citations per paper (106.95) and IF (2020, 7.194), occupying an important position in this field. The IF of these journals mainly concentrated in the range from 2 to 4, revealing that relevant papers published in high-IF journals present great challenges. Pearson correlation analysis revealed that publication outputs and IF had no significant correlation (*r* = −0.198, *p* = 0.584). In Journal Citation Reports, the majority of the top 10 journals belonged to the *Rehabilitation* category and were in Q1 (Table 1).

**TABLE 1:** Top 10 journals based on publication outputs.

Rank	Journal	Papers	Country	Citations (WoS)	Citations per paper	IF (2020)	IF (5 years)	JCR categories	Quartile
1	Journal of Physical Therapy Science	175	Japan	1,880	10.74	0.392 (2014)	0.414	REHABILITATION (SCIE)	Q4
2	Neurorehabilitation and Neural Repair	160	United States	7,159	44.74	3.919	5.378	CLINICAL NEUROLOGY; REHABILITATION (SCIE)	Q2; Q1
3	Clinical Rehabilitation	145	United Kingdom	5,250	36.21	3.477	4.193	REHABILITATION (SCIE)	Q1
4	Archives of Physical Medicine and Rehabilitation	127	United States	7,432	58.52	3.966	4.489	REHABILITATION (SCIE); SPORT SCIENCES	Q1; Q2
5	Topics in Stroke Rehabilitation	125	United States	1,767	14.14	2.119	2.797	REHABILITATION (SCIE)	Q3
7	Journal of Neuroengineering and Rehabilitation	112	United Kingdom	3,447	30.78	4.262	5.218	ENGINEERING, BIOMEDICAL; NEUROSCIENCES; REHABILITATION (SCIE)	Q2; Q2; Q1
6	Stroke	108	United States	11,551	106.95	7.194	8.032	CLINICAL NEUROLOGY; PERIPHERAL VASCULAR DISEASE	Q1; Q1
8	Journal of Stroke Cerebrovascular Diseases	96	United States	875	9.11	2.136	2.185	NEUROSCIENCES; PERIPHERAL VASCULAR DISEASE	Q4; Q4
9	Neurorehabilitation	83	Ireland	891	10.73	2.138	2.501	CLINICAL NEUROLOGY; REHABILITATION (SSCI); REHABILITATION (SCIE)	Q4; Q2; Q3
10	Disability and Rehabilitation	77	United Kingdom	1,127	14.64	3.033	3.298	REHABILITATION (SSCI); REHABILITATION (SCIE)	Q1; Q1

**FIGURE 4:**
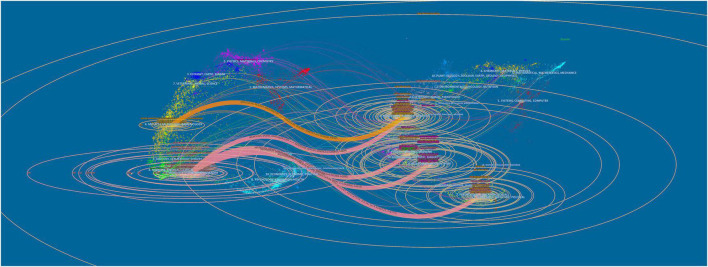
The dual-map overlay of journals.

Figure 4 shows a dual-map overlay of all academic journals, mapping the citation paths of subject fields. The labels on the left of the dual-map represent the discipline covered by the citing journals, whereas those on the right indicate the discipline of the cited journals. Most journals were from neurology, sports, ophthalmology, medicine, medical, and clinical field, called research frontier. Cited papers were mainly published in molecular, biology, genetics, health, nursing, medicine, sports, rehabilitation, psychology, education, and social journals, called knowledge base.

### Author Analysis

**FIGURE 5:**
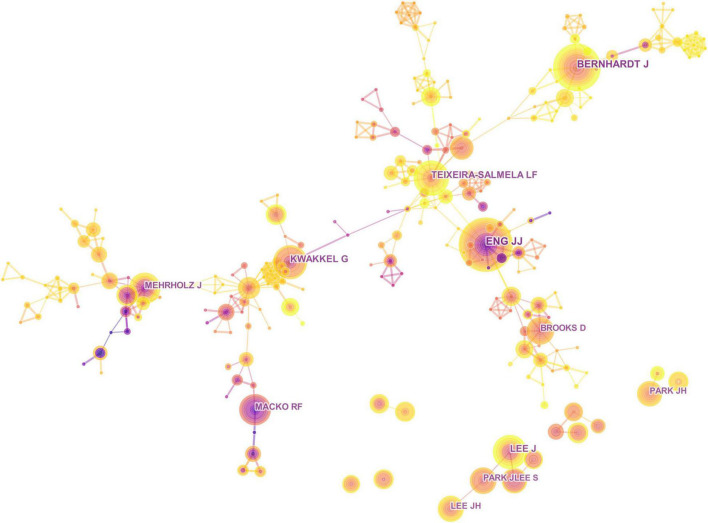
Collaboration network of authors.

A total of 12,130 authors published these papers. Figure 5 shows the collaboration network of authors. Amongst the top prolific authors, Eng J. J. ranked the first (36 publications), followed by Bernhardt J. (34 publications), Kwakkel G. (29 publications) and Teixeira-Salmela L. F. (29 publications). Eng J. J. was based at the University of British Columbia. He led their research team to design the Graded Repetitive Arm Supplementary Program that helped improve the recovery of stroke survivors’ arm function and conducted a study on the use of robotic exoskeleton for walking training early after stoke. However, the link between nodes was sparse, and the centrality for cooperation was 0, indicating that the cooperation between various authors in this field was not very sufficient.

### Keyword Co-occurrence

**FIGURE 6:**
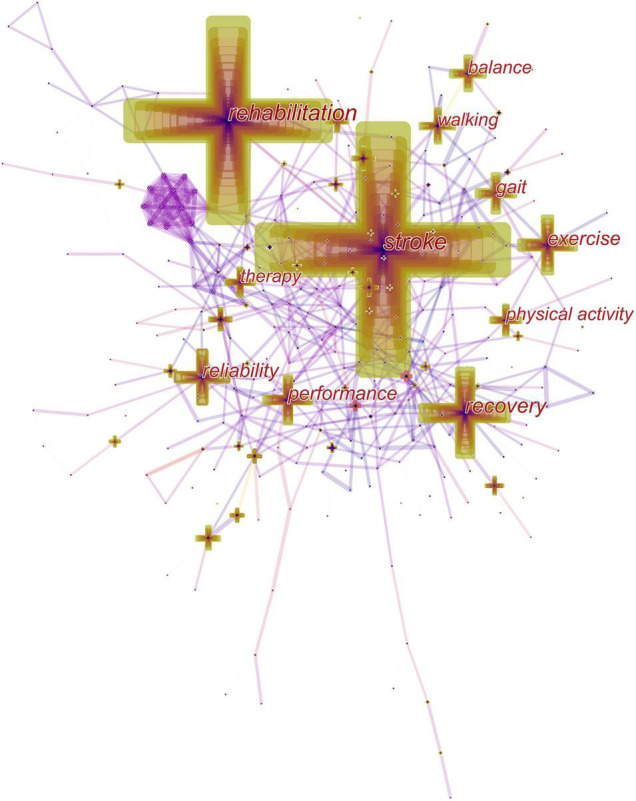
Co-occurrence network of keywords.

**FIGURE 7:**
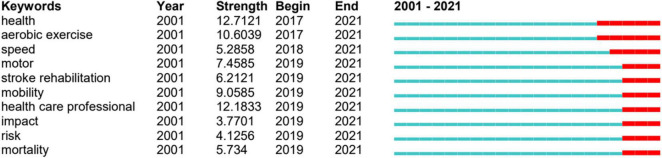
Burst keywords with frequent citations until 2021.

Keywords with high frequency of occurrence and high value of centrality reveal the research hotspots in the past 20 years, and those with strong citation bursts can predict future research frontiers. By the end of 2021, “stroke,” “rehabilitation” and “recovery” were the top three high-frequency keywords. In terms of centrality, “brain” ranked the first, followed by “activation” and “mechanism” (Figure 6). The top 40 keywords obtained based on the times of occurrence and centrality were divided into eight themes (diseases, treatment, body region, outcome, evaluation, population, research design, and mechanism) (Table 2). Amongst the burst keywords, “health,” “aerobic exercise,” “speed,” “motor,” “stroke rehabilitation,” “mobility,” “health care professional,” “impact,” “risk,” and “mortality” were cited frequently until 2021 (Figure 7).

**TABLE 2:** Research keywords by theme.

Theme	Keywords
Disease	Stroke, impairment, hemiparesis, cerebrovascular accident, coronary artery disease, lesion, cerebrovascular disorder
Treatment	Exercise, gait, walking, therapy, physical activity, balance, physical rehabilitation, strength, locomotion
Body region	Upper extremity, upper limb, arm, activation
Outcome	Rehabilitation, recovery, performance, follow-up, quality of life, mortality
Evaluation	Reliability, validity
Population	Individual, people
Research design	Randomized controlled trial
Mechanism	Brain, cortex, mechanism, cortical reorganization, reorganization, plasticity

### Reference Co-citation

**FIGURE 8:**
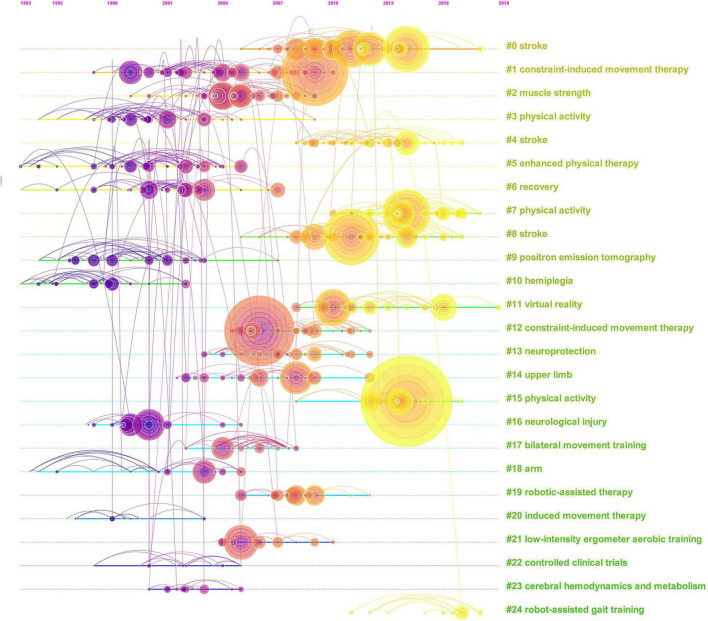
Timeline view of references.

A timeline map of co-citation references with 19 clusters is shown in Figure 8. Each cluster was composed of papers with similar topics, named by nominal terms extracted from keywords in these co-cited papers. Cluster #0 (plasticity) was the largest, followed by cluster #1 (rct-rehabilitation-stroke interventions-upper extremity-motor recovery-behavior) and cluster #2 (sensorimotor impairments). Cluster #18 (movement training) appeared the earliest. Cluster #3 (physical activity) gradually attracted people’s attention in recent years. The most cited reference (physical activity and exercise recommendations for stroke survivors: a statement for healthcare professionals from the American Heart Association/American Stroke Association) belonged to cluster #3 (physical activity) (Table 3).

**TABLE 3:** The top five references based on the number of citations.

Rank	Title	First author	Corresponding author	Document type	Year	Country	Journal	IF (2020)	Citations (WoS)
1	Physical activity and exercise recommendations for stroke survivors: a statement for healthcare professionals from the American Heart Association/American Stroke Association	Billinger, S. A.	Billinger, S. A.	Review	2014	United States	Stroke	7.914	577
2	Effect of constraint-induced movement therapy on upper extremity function 3 to 9 months after stroke: the EXCITE randomized clinical trial	Wolf, S. L.	Wolf, S. L.	Article	2006	United States	JAMA	56.274	1,211
3	Motor recovery after stroke: a systematic review	Langhorne, P.	Langhorne, P.	Review	2009	United Kingdom	Lancet Neurology	44.182	1,053
4	Stroke Care 2 Stroke rehabilitation	Langhorne, P.	Langhorne, P.	Article	2011	United Kingdom	Lancet	79.323	1,161
5	What is the evidence for physical therapy poststroke? A systematic review and meta-analysis	Veerbeek, J. M.	Kwakkel, G.	Review	2014	Netherlands	PLoS One	3.24	519

### Landmark Publication

Table 4 demonstrates the top five publications with the highest citation frequency. The paper by Wolf et al. (Effect of constraint-induced movement therapy on upper extremity function 3 to 9 months after stroke: The EXCITE randomized clinical trial) was the most cited. It was published in JAMA and had relatively high average annual citations and the highest IF. The top two papers in Table 4 were also the top two references in Table 3. A majority of papers had the same first author and corresponding author and were published before 2010.

**TABLE 4:** The top five papers based on the number of citations.

Rank	Title	First author	Corresponding author	Document type	Year	Country	Journal	IF (2020)	Citations (WoS)	Citations per year
1	Effect of constraint-induced movement therapy on upper extremity function 3 to 9 months after stroke - The EXCITE randomized clinical trial	Wolf, S. L.	Wolf, S. L.	Article	2006	United States	JAMA	56.274	1,211	134.07
2	Primary prevention of ischemic stroke – a guideline from the American Heart Association/American Stroke Association stroke council: cosponsored by the atherosclerotic peripheral vascular disease interdisciplinary working group; cardiovascular nursing council; clinical cardiology council; nutrition, physical activity, and metabolism council; and the quality of care and outcomes research interdisciplinary working group – the American academy of neurology affirms the value of this guideline	Goldstein, L. B.	/	Review	2006	/	Stroke	7.914	756	50.40
3	Robot-assisted movement training compared with conventional therapy techniques for the rehabilitation of upper-limb motor function after stroke	Lum, P. S.	Lum, P. S.	Article	2002	United States	Archives of Physical Medicine and Rehabilitation	3.966	712	37.47
4	Effects of augmented exercise therapy time after stroke – A meta-analysis	Kwakkel, G.	Kwakkel, G.	Review	2004	Netherlands	Stroke	7.194	646	38.00
5	Physical activity and exercise recommendations for stroke survivors a statement for healthcare professionals from the American Heart Association/American Stroke Association	Billinger, S. A.	Billinger, S. A.	Review	2014	United States	Stroke	7.914	574	82

In summary, the number of publications related to this research keeps increasing every year. Different countries have shown different international influences in this research field. The results also revealed some core journals and potential collaborators. The analysis of keywords and references indicated research status and hot spots, as well as emerging trends.

## Discussion

### Global Trends of Exercise for Stroke Research

To our knowledge, this is the first bibliometric analysis of exercise for stroke based on WoSCC. We used the CiteSpace tool to obtain 3,484 published papers and show the continuous scholarly work in this field of exercise for stroke between 2001 and 2021. The results show that the number of publications increased gradually annually. The rapid growth of publications does not indicate that these papers have high quality; it only indicates that many researchers pay more attention to exercise for stroke. The top 10 journals did not have high IF. The journals with extremely high IF, such as Lancet, Nature did not publish the topic articles. Maybe, we think that this field has some innovative and breakthrough discoveries. According to the results, the United States has a positive sense of collaboration and plays an important role in the field of exercise for the research. There was strong positive correlation between the GDP of countries and stroke publication output (Salhab et al., 2018). We think that the strong economic foundation and larger number of stroke survivors have endowed the United States with important role in the research.

### Research Focuses on the Exercise for Stroke Research

According to the co-occurrence network of keywords, the keywords with the highest frequency are “stroke,” “rehabilitation,” and “recovery”; that is to say, how to recover or rehabilitate the patients with stroke is still a hot topic in the field of exercise for stroke. Figure 7 shows that the burst keywords were detected by CiteSpace V. According to these data, we could know the frontiers of exercises for the research. The top burst keywords are “health,” “aerobic exercise,” and “speed,” indicating potentially emerging trends. The three research trends are as follows:

1. Health: there are different kinds of interventions for different health problems in stroke survivors. Physical exercises combined with cognitive training produced greater benefits on cognitive function in survivors with vascular cognitive impairment (Johnson et al., 2018; Bo et al., 2019). Chinese traditional exercises could help reduce symptoms of anxiety and stress and improve the sleep of the patients with stroke (Taylor-Piliae et al., 2021). Stroke was closely related to cardiovascular health; the effect of intensity and duration of exercise on cardiorespiratory fitness in patients with stroke could be a good topic in future research (Langhammer et al., 2014; Johnson et al., 2018; Luo et al., 2020).
2. Aerobic exercise: aerobic exercise not only has a positive effect on mood, quality of life, and aerobic capacity in patients with early stroke, but also changes the metabolomic profiles in patients with chronic stroke (Pang et al., 2013; Gezer et al., 2019). The evidence and mechanism of the effect of aerobic exercise in patients with stroke have been studied. For example, moderate forced exercise could reduce lesion volume and protect perilesional tissue against oxidative damage and inflammation in early-initiated (24–48 h post-stroke) (Austin et al., 2014).
3. Speed: walking speed can predict and influence functional recovery in patients with stroke. For example, walking speed exercise can improve cognitive function in patients with chronic stroke (Kim and Yim, 2017). Aerobic exercise has a positive effect on gait speed in individuals who have had a left-side stroke (Catapani et al., 2021). Walking speed could be used as an evaluation index and exercise protocol for stroke survivors. There is no mechanistic measure to explain the changes in walking speed during neurologic recovery. A multi-modal approach is necessary for stroke rehabilitation (Wonsetler and Bowden, 2017).

According to the top five references based on the number of citations, we can determine the potentially useful references for exploring the knowledge base of research frontiers. The American Heart Association’s Stroke Council’s Scientific Statement Oversight Committee and Manuscript Oversight Committee provided that the management of stroke survivors could benefit from physical activity and exercise prescription in daily life (Billinger et al., 2014). Many kinds of trials showed that a broad range of interventions could help stroke survivors to recover movement and related function, including constraint-induced movement therapy, electromyographic biofeedback, mental practice with motor imagery and robotics (Langhorne et al., 2009). Patents with different kinds of stroke need different exercise therapy. For example, amongst patients who had their first stroke 3–9 months previously, constraint-induced movement therapy could be an effective method to improve paretic arm function (Wolf et al., 2006). Physical therapy poststroke and intensive high repetitive task-oriented and task-specific training can be used for all phases poststroke (Veerbeek et al., 2014). Therefore, we think that the time point of recovery needs to be focused in the future research, and lager clinical trials with sufficient statistical power are needed to be obtained (Langhorne et al., 2011).

### Strengths and Limitations

This is the first bibliometric analysis to summarize the progress and trends of exercise for stroke in the past 20 years on the basis of WoS data. To gain integrated and diverse data, our study included 3,484 publications, and these papers were published in 584 academic journals. Furthermore, this bibliometric analysis not only contained publications, journals, citations, collaboration network of authors, countries, and regions; it also included burst keywords, research keywords by theme, co-occurrence network of keywords, and the top five references and papers on the number of citations.

However, this study has some limitations. Firstly, our study did not search other academic databases, such as PubMed, Embase, and Cochrane Library. Secondly, the variety of languages was limited. The study selected English as the inclusion criterion, papers with other languages were excluded. This could result in publication bias. Thirdly, there was no accurate identification and separation of the interconnections amongst authors, regions or countries, even though our study analyzed their collaboration network.

## Conclusion

This study analyzed the trends of exercise for stroke and provided the potential research frontiers in the past 20 years, it can prove useful as a basis for developing improved exercise interventions for stroke. The topic of exercise interventions for stroke is becoming more and more popular amongst clinical workers and researchers. This analysis may enable research teams to collaborate in promoting the application of exercise therapy in the clinical management of stroke. Although this study has some limitations, it provides a useful basis for further research into hot topics, research focuses, cooperators, and development trends of exercise for the research.

## Author Contributions

XW and JW: research concept and design. LW and YD: collection and assembly of data and writing the manuscript. TS and ZL: data analysis and interpretation. WW, YZ, and RD: critical revision of the manuscript. YH, YM, and JW: final approval of the manuscript. All authors have read and approved the manuscript.

## Conflict of Interest

The authors declare that the research was conducted in the absence of any commercial or financial relationships that could be construed as a potential conflict of interest.

## Publisher’s Note

All claims expressed in this article are solely those of the authors and do not necessarily represent those of their affiliated organizations, or those of the publisher, the editors and the reviewers. Any product that may be evaluated in this article, or claim that may be made by its manufacturer, is not guaranteed or endorsed by the publisher.
